# Editorial: African Cultural Models in Psychology

**DOI:** 10.3389/fpsyg.2022.849622

**Published:** 2022-05-11

**Authors:** Robert Nicholas Serpell

**Affiliations:** Psychology Department, University of Zambia, Lusaka, Zambia

**Keywords:** Africa, linguistic relativity, cultural models, cultural hegemony, biculturation

## Introduction

The focus of this Research Topic was defined by the Editors as “*cultural models* that describe or explain patterns of existing within, making sense of, interacting with, and shaping the world that stem from African experiences,” and they expressed the hope that it would help to “better represent Africa in Psychology to make the discipline less WEIRD.” Several elements of that broad goal were addressed in different ways by the various studies in the collection. How should Africa be represented? Cultural models are a form of collective representation shared among member-owners of a particular culture (Quinn and Holland, 1987). How should they feature in systematic research on psychological topics? As hypothetical influences on individual behavior and experience? As frameworks for data collection? Or as thematic resources for communication between various social groups? And how should they be attributed? To a geopolitical region, such as Africa? Or to an ethnolinguistic group, such as indigenous members of a community…

- with attention to sociocultural change over time (e.g., 1970s vs. 2010s)?
- with attention to ascribed authority (e.g., insiders, elders, respected local experts)?
- as contestable interpretations (e.g., which traditional practices deserve enduring loyalty while others are obsolete?)

Answering those conceptual questions has implications for both methodology and theory. All the peoples on earth face the same global catastrophes of climate change and pandemics. Moreover, as members of a single biological species, we share many dispositions and vulnerabilities. Yet we draw on remarkably different cultural traditions to interpret the mysteries of the human condition, and those explanations of the unobservable are incorporated into the ways each generation raises its young. A common theme across most of the studies in this collection is that the contemporary mainstream of psychology tends to address theoretical issues with insufficient attention to the cultural history of how the discipline has emerged. Despite that very particular historical origin, much of the psychological research published in international journals takes for granted cultural preferences shared among Western authors and their audiences that are not necessarily shared by outsiders (Serpell, 1994). The founders of modern science claimed that their Enlightenment (a new, more reliable understanding of the world) was derived from methods of investigation that are open to others to try out for themselves. That method-grounded character of modern science sets it apart from other systems of knowledge such as the major world religions. But it has acquired ideological overtones that sometimes command loyalty beyond its rational explanatory power. Tensions can arise between scientific researchers, policymakers and other stakeholders in the application of research evidence to public policy and practice around the relative status of scientific knowledge and other ways of knowing. Strategic consensus appears to be emerging in academia that understanding psychological phenomena demands paying systematic attention to sociocultural context. This collection illustrates a wide variety of methods for doing so, including ethnography, purposive sampling, and statistical control of demographic variables.

## Seven Studies Representing Diversity Within the African Continent

Table 1 summarizes some relevant descriptive features of the seven studies: the country in which the research was conducted, the demographic profile of the population sampled, and the languages invoked by the study for data collection and/or interpretation. Nation does not always coincide in a simple way with ethnolinguistic group. For instance, the meta-analysis of research in Africa on intelligence authored by Oppong, a thinker of Ghanaian origin, residing and writing in Botswana, draws evidence for an African perspective from three studies conducted by different researchers in Zambia (in the 1970s and 80s), in Kenya (in the 1990s) and in Togo (in the 2000s). His systematic review selected those three studies from a preliminary sample of 1770 records published after 1990, on the basis of various theoretical considerations, including an account of “African cultural values” by the Ghanaian professor of philosophy, Kwame Gyekye. He concludes that an African model of valued human cognitive ability should expand the scope of Carroll's (1993) three-stratum theory of intelligence to include wisdom and socioemotional competence. While this might seem to be grounded in a rather small sample of empirical studies, it appears to me consistent with much of the other relevant research literature.

**Table 1:** Descriptive features of the studies.

**Short title**	**Human cognitive abilities**	**Models of wellbeing**	**Relationships as sources of meaning**	**Women empowerment**	**Validating indigenous versions of personality inventory**	**Self-determination of healthy eating**	**Dilemma tales**
Author(s)	Oppong	Osei-Tutu et al.	Wissing et al.	De Smet and Boros	Hill et al.	De Man et al.	Esiaka et al.
Psychological domain	Intelligence	Subjective wellbeing	Subjective wellbeing	Gender relations	Personality assessment	Personal agency	Moral reasoning
Country (ies)	Zambia, Kenya, Togo	Ghana	Ghana, South Africa	Ethiopia (SNNPR)	South Africa	South Africa	Ghana, USA
Demographic sample (s)	Rural adults	Adult indigenous language native speakers	Urban and rural adults	Rural adults	Adult indigenous language native speakers	Urban adults	Urban adults
Language (s)	Chewa, Luo, Ewe/Gengbe	Akan, Ewe, Ga, Dagbani	English, Tswana	Amharic	Venda, Sotho, English	Xhosa	English
(African language family)	Niger-Congo (Bantu) Nilo-Saharan (Nilotic) Niger-Congo (Kwa)	Niger-Congo (Kwa) Niger-Congo (Gur)	Niger-Congo (Bantu)	Afro-Asiatic	Niger-Congo (Bantu)	Niger-Congo (Bantu)	unspecified

The sociocultural diversity of the African region sometimes appears to defy collective representation. The studies in this collection were conducted in <10 of the 52 member states of the African Union and, drew on <20 of the continent's more than 1,500 recognized languages. But it is noteworthy that the languages sampled are spread across the four language families identified by linguistics as most widely spoken in various parts of the continent: Niger-Congo, Nilo-Saharan, Afro-Asiatic and Indo-European. The study by Osei-Tutu et al. of indigenous models of wellbeing across different languages within the nation of Ghana is complemented by Wissing et al.'s study of relationships as sources of meaning across Ghanaian and South African societies.

The Ghanaian study explored in conversation with adult native speakers of Akan, Ewe, Ga or Dagbani the vocabulary of each language related to the domain of subjective wellbeing, and identified four implicit themes across all the languages, about how wellbeing is conceptualized. Two of the themes, good health and positive affect, appeared to “resonate with standard understandings of wellbeing in < what the authors term> hegemonic psychological science,” while two others (morally, materially or relationally) good living and peace of mind, seemed to reflect a sustainability or maintenance orientation to wellbeing that differs significantly from a “standard” understanding, that emphasizes individual psychological fulfillment and high-arousal positive affect. The authors thus rightly question whether that “standard” is self-evident to audiences and clients across the world. If the promotion of wellbeing by international organizations such as the World Health Organization (WHO) is to be responsive to cultural preoccupations of all human societies, it will need to be broadened beyond the particular themes dominant in Western cultural-linguistic groups, such as self-realization and happiness.

The principle of attributing authoritative knowledge of the meaning-system of a language to its “native-speakers” seems uncontroversial, but, as the authors acknowledge, “it is not clear how different cultural models of wellbeing manifest in language map onto individual subjective experience of wellbeing.” The classic linguistic relativity hypothesis has received only limited support from systematic research (Gumperz and Levinson, 1991). Moreover, given the prevalence of individual plurilingualism in Africa, it seems likely that, even within one “language group,” the evidence for ownership of a conceptual model implicit in its vocabulary must include the likelihood that individuals have a multi-stranded repertoire, which they deploy in accordance with situational and diachronically changing parameters, permeated by issues of power. Programmatic social interventions such as vaccination, schooling and banking involve interactions, not only between different social groups (professionals, politicians, etc.) but also among various strands of a single individual's cognitive repertoire (biomedical knowledge, common sense, religious faith, etc.) that influence personal decision-making such as whether to receive a vaccine injection, or whether to enroll one's child in a local early childhood care facility.

The two-nation study of subjective wellbeing by-passes such complexities, entrusting to backtranslation the responsibility for establishing equivalence between English and Setswana for the highly abstract focal request for the informant to list “things that you consider most meaningful in your present life.” But it is not clear that linguistic translation can be isolated in that way from the psychological focus of the study on “what provides meaning in life” and how. For instance, in the cognate Southern Bantu language chiChewa, the four strongest candidate terms to translate *meaningful* (*thantauzo, pindula, thandiza*, and *mveka*) (Paas, 2016) might also translate back into English, respectively, as *significant, profitable, helpful*, or *understood*. Choosing one of these as the elicitation cue, while not arbitrary, would likely tend to bias responses in a particular direction.

Diversity of socioeconomic, educational and residential status is also illustrated in several of the studies, and the importance of intersectionality is addressed by the study of gender relations in Ethiopia. All of those dimensions of variation are relevant to understanding “patterns of existing within, making sense of, interacting with, and shaping the world that stem from African experiences.” Somewhat less clearly articulated are the phenomena of individual biculturation and developmental change. A science of psychology that represents comprehensively the African condition will need to pay explicit attention to both, and to the cultural practices and beliefs of minority groups such as the indigenous hunter-gatherer communities inhabiting various zones of the continent (e.g., Lew-Levy et al., 2019). Arguably the cultures of exogenous settler and colonizing groups already receive disproportionate attention in many facets of social science in Africa, and have been over-represented in the psychological literature to date by selecting convenience samples of university students and citing them spuriously as guarantors of local cultural validity. A decision by cultural psychologists to focus elsewhere is a legitimate research strategy for counter-hegemonic discourse, but the cultural models favored by elites will eventually need to be part of a comprehensive representation of Africa.

The study of gender relations in Ethiopia raises a challenging set of issues in that regard. The authors, De Smet and Boroş, who declare their own cultural identity as Europeans, initiated the project as part of a participatory women empowerment intervention. Reflecting on the limited sustainability of the intervention, they explain how well-intentioned researchers from the global north can misinterpret cultural resistance as failure by the host community to understand the authors' methodology. A key principle of the intervention, termed “facilitation from the back,” “is based on the assumption that change comes by giving equal voice to all people who are part of the process. Consequently, the method is designed to challenge existing power inequalities by everybody participating in an equal way” (Mayoux, 2017). This egalitarian perspective was operationalized in a nuanced way in the Participatory Action Learning System (PALS) methods used to launch their project. The authors interpret the failure of local Ethiopian NGO staff to adopt such an egalitarian approach in carrying the project forward as a case of “micro-resistance” to a foreign imposition inconsistent with their own cultural values. Yet, this study is silent on the sustainability of those values in a changing world. It would be interesting to learn how socially progressive Ethiopian women would justify adherence to their traditional modes of communication. The European authors of the study, drawing on Hofstede's (1984) hologeistic theory, describe Ethiopian society as an exemplar of a *tight* culture high in *power-distance*, in which deviations from local norms are strictly prohibited. Accepting this exogenous characterization of their society would seem to portray the community as politically deficient with respect to possibilities of progressive social change. But it seems likely that the Ethiopian women staff participants appropriated the project's empowerment agenda differently through their own cultural lens.

The South African study of indigenous personality assessment by Hill et al. builds on the ambitious, long-term programme initiated by Fetvadjiev et al. (2015) to develop a cross-culturally applicable South African Personality Inventory (SAPI). A key feature of the SAPI development project has been to work from the bottom upwards, starting with qualitative (emic) data collection and building up to quantitative (etic) measurement. Candidate items were initially generated through interviews to obtain personality descriptors of familiar persons from adult first-language speakers of each of the 11 official language groupings in South Africa. Conceptual and hierarchical analysis of these descriptors resulted in nine personality clusters, six of which were interpreted as representing agentic or personal-growth elements, while the other three are more communal or social-relational. This new study translated the SAPI into two of the South African official languages, Tshivenda and Southern Sotho, and used Exploratory Structural Equation Modeling to analyze the data. The overall fit of the six-factor model was excellent, and most facets loaded acceptably on their expected factors. However, the language in which the inventory was completed influenced the specific factor structure that emerged, and unexpected relations were found among several facets. Moreover, the facet of traditionalism-religiosity, considered especially important by indigenous African respondents, was found to be inadequately represented by the four items devoted to it. The authors concluded that the different patterns of responses elicited by these indigenous language versions of the inventory “are to some extent justifiable based on the initially identified implicit perspectives on personality of the two language groups” (p. 11).

Reviewing the range of methods available in South Africa for psychological assessment that are appropriate and fair for clients from diverse cultures, Laher and Cockcroft (2017, p. 3–4) concluded that “the best assessment practice for multiculturally diverse contexts is the development of emic (culturally and linguistically specific to a particular context) measures, rather than relying on adaptations of existing tests developed for other contexts and communities.” That appraisal seems consistent with the “final goal of the combination of emic and etic approaches” stated by the originators of the SAPI as helping to “overcome the dichotomous view of personality traits as either culture-specific or universal and give way to a more gradual view of levels of universality and cultural specificity of traits” (Nel et al., 2012, p. 945). Rather than aiming for a cross-culturally universal characterization of individual differences, a more appropriate goal may be to acknowledge that a plurality of standards is relevant to the assessment of behavior in identifiably different socio-cultural contexts.

Another South African study of personal agency in dietary behavior by diabetes patients by De Man et al. adopts an unabashedly top-down approach to local validation of a universalistic (some might say hegemonic) theory of self-determination. A sample of overweight residents in a low-income periurban settlement was recruited who had been biomedically diagnosed as at risk for, or living with, diabetes. A self-report questionnaire comprising a set of multiple-choice questions derived from empirical research elsewhere was translated into the local language, isiXhosa, and administered to assess their selfperception, perceived social support, motivation, and consumption of specific food groups as an indicator of healthy diet. Structural Equation Modeling was used to test the theoretical model and confirmed that, over and above perceived competence and social support for dieting, the kind of motivation expressed by respondents was predictive of their dietary behavior. Patients who emphasized their own personal responsibility showed healthier dietary consumption than those who emphasized compliance with social pressure. This study provides impressive replication of a pattern of findings in other socio-cultural contexts about adherence to medical advice for the prevention and treatment of a significant adverse condition of physical health. However, due to the tightly specified top-down operationalization of the measures administered, it is silent about the possible relevance of factors distinctive to the eco-cultural context that might be less influential elsewhere. Are there, for instance (as documented by Chakona and Shackleton, 2019 in a neighboring province) any widely respected beliefs or practices in this community that compete with the prescriptions of modern cosmopolitan medicine?

The study of moral reasoning by Esiaka et al. is unique in this collection, both in including an African-American sample and in invoking an indigenous African discourse practice as a framework for eliciting evidence of participants' beliefs. The positing of a moral dilemma within a fictional story is, according to these authors, a widespread informal discourse practice across several African nations, including Ghana, and regarded as a long-standing part of the indigenous culture, that affords both entertainment and opportunities for reflection and debate about relational norms. They adapted the dilemma tale format to create a questionnaire, administered in English to three adult samples with at least high school education: Ghanaian (non-student) residents on the campus of the national university campus, African-Americans and European-Americans (the latter two groups recruited through an online marketplace). The authors' interpretation of their substantive findings in this study center on the popular binary contrast in cross-cultural psychology between collectivist and individualist cultures. As predicted from that contrast, European-American respondents prioritized obligations to their spouse over their mother more than Ghanaians or African-Americans. But African-Americans responded more like European-Americans with respect to endorsing institutional elder care, perhaps reflecting the greater ecological availability of quality elder care in the USA than in Ghana. The complexity of factors influencing attitudes expressed in response to these intriguing but somewhat unrealistic dilemma tales was further revealed by attention in the analysis to respondents' developmental experience of family relationships and current residential settings. It seems then that a full explanation of an individual's (hypothetical) decision-making about allocation of household resources calls for not only an appreciation of a cultural model shared with other members of their ethnocultural reference group, but also of their current ecological setting and their past developmental experience.

## Discussion: Ways Forward

This rich collection of studies contributes to an understanding of multiple ways in which African societies can benefit from application of psychological science. A working hypothesis of the Editors was that indigenous African cultures afford models that have been neglected to date and could enrich psychology. Because the discipline has been dominated from its inception by thinkers whose primary enculturation was in WEIRD societies, the relevance of African cultural models is only just beginning to be appreciated (Thalmayer et al., 2021). Two types of strategic approach are exemplified in this collection: starting at the top with abstract theoretical ideas conceived as universal and working downwards from there to test their relevance and limitations in Africa; or starting at the bottom with intuitions of socioculturally situated African participants and working upwards in search of integrative interpretations for comparison or merger with those derived from research in WEIRD societies. The emic-etic methodology invoked by developers of the SAPI names rather than explaining this dichotomy. The specter of Western cultural hegemony features in the studies adopting a top-down strategy and those characterizing African societies in terms of a hologeistic typology of cultures. Conversely, the bottom-up strategy evokes the limitations of the linguistic relativity perspective and the dangers of premature generalization. The connection between social and psychological representations is theoretically complex, and language is just one of several relevant dimensions of human sociocultural diversity. Each of the psychological domains addressed in these studies requires explicit theoretical articulation, and the advent of structural equation modeling affords a new degree of precision for doing so in measurable ways open to stringent empirical evaluation. But, if the goal of generating a less WEIRD psychology is to be attained, qualitative exploration of the categories that make sense to local African audiences will be an essential prerequisite to valid quantitative investigations.

## Author Contributions

The author confirms being the sole contributor of this work and has approved it for publication.

## Conflict of Interest

The author declares that the research was conducted in the absence of any commercial or financial relationships that could be construed as a potential conflict of interest.

## Publisher's Note

All claims expressed in this article are solely those of the authors and do not necessarily represent those of their affiliated organizations, or those of the publisher, the editors and the reviewers. Any product that may be evaluated in this article, or claim that may be made by its manufacturer, is not guaranteed or endorsed by the publisher.

## References

[B1] Carroll J. B. (1993). Human Cognitive Abilities: A Survey of Factor-Analytic Studies. New York, NY: Cambridge University Press. 10.1017/CBO9780511571312

[B2] Chakona G. Shackleton C. (2019). Food taboos and cultural beliefs influence food choice and dietary preferences among pregnant women in the Eastern Cape, South Africa. Nutrients 11, 2668. 10.3390/nu11112668 31694181 PMC6893604

[B3] Fetvadjiev V. H. Meiring D. Van de Vijver F. J. Nel J. A. Hill C. (2015). The South African Personality Inventory (SAPI): a culture-informed instrument for the country's main ethnocultural groups. Psychol. Assess. 27, 827. 10.1037/pas0000078 25602691

[B4] Gumperz J. J. Levinson S. C. (Eds) (1991). Rethinking linguistic relativity. Curr. Anthropol. 32, 613–23. 10.1086/204009

[B5] Hofstede G. (1984). Culture's Consequences: International Differences in Work-Related Values. Beverly Hills, CA: Sage.

[B6] Laher S. Cockcroft K. (2017). Moving from culturally biased to culturally responsive assessment practices in low-resource, multicultural settings. Profess. Psychol. Res. Pract. 48, 115–121. 10.1037/pro0000102

[B7] Lew-Levy S. Crittenden A. N. Boyette A. H. Mabulla I. A. Hewlett B. S. Lamb M. E. (2019). Inter-and intra-cultural variation in learning-through-participation among Hadza and BaYaka forager children and adolescents from Tanzania and the Republic of Congo. J. Psychol. Afr. 29, 309–318. 10.1080/14330237.2019.1647957

[B8] Mayoux L. (2017). “*PALS Toolkit: SNV Ethiopia.” GAMEchange Network, Comic Relief* . Available online at: https://gamechangenetwork.org/?s=Mayoux (accessed May 3, 2022).

[B9] Nel J. A. Valchev V. H. Rothmann S. Van de Vijver F. J. Meiring D. De Bruin G. P. (2012). Exploring the personality structure in the 11 languages of South Africa. J. Pers. 80, 915–948. 10.1111/j.1467-6494.2011.00751.x 22091948

[B10] Paas S. (2016). Oxford ChiChewa-English Dictionary, 5th Edn. Cape Town: Oxford University Press.

[B11] Quinn N. Holland D. (1987). Cultural Models in Language and Thought. Cambridge University Press. 10.1017/CBO9780511607660

[B12] Serpell R. (1994). Negotiating a fusion of horizons: a process view of cultural validation in developmental psychology. Mind Cult. Activity 1, 43–68. 10.1080/10749039409524656

[B13] Thalmayer A. G. Toscanelli C. Arnett J. J. (2021). The neglected 95% revisited: is American psychology becoming less American?. Am. Psychol. 76, 116. 10.1037/amp0000622 32271027

